# Multiple criteria decision making and robust optimization to design a development plan for small and medium-sized enterprises in the east of Iran

**DOI:** 10.1007/s12351-023-00761-1

**Published:** 2023-02-25

**Authors:** Sahar Moazzeni, Sobhan Mostafayi Darmian, Lars Magnus Hvattum

**Affiliations:** 1 Department of Business and Management Science, NHH Norwegian School of Economics, Bergen, Norway; 2 Department of Mechanical and Industrial Engineering, NTNU, Trondheim, Norway; 3 Faculty of Logistics, Molde University College, Molde, Norway

**Keywords:** Industrial cluster, Governmental funding, Multi-objective optimization

## Abstract

This paper is based on a project of developing small and medium-sized enterprises in South Khorasan, Iran. A combination of multiple criteria decision making and robust multi-objective optimization is used for prioritizing industrial clusters and optimally assigning governmental funds. First, criteria for evaluating clusters are weighted using the best-worst method and clusters are prioritized using the VIKOR method. Second, governmental funding is assigned to the highest priority cluster using robust multi-objective mathematical programming. An innovative method is applied to identify the solution of the Pareto-front with the highest efficiency. In a case study from South Khorasan, computational results show that international market share attraction is the most important criterion and the age of the cluster is the least important criterion in the development of the industrial clusters. The cluster defined for barberry and jujube fruits is determined to be the first-ranked cluster. Most of the total available budget for the development of this cluster is assigned to action plans for marketing and trade development and for investment and financial planning. The proposed methodology thus successfully aided the decision makers to plan their work regarding funding of industrial clusters. We believe that the methodology can be applied as a general tool to help managers of industrial development to better assign governmental funding to develop industrial clusters.

**Supplementary Information:**

The online version contains supplementary material available at 10.1007/s12351-023-00761-1.

## Introduction

In the last decade, managers in various sectors of agriculture, economy, and industry have considered the development of small and medium-sized enterprises (SMEs) as a key strategy to increase economic growth and to improve the business environment (McCann and Ortega-Argilés 2016). Different policies have been proposed to execute development programs for SMEs, and the introduction of industrial clusters have led to more desirable results than other policies (Garone et al. 2015). In general, industrial clusters include horizontal, vertical, and lateral connections between activities related to value creation on product groups using a set of economic, political, environmental, and social factors (McCann and Ortega-Argilés 2016). The boundaries of cluster activities are determined by direct and indirect interactions between the factors that create weak, moderate, and strong links between cluster members (Hu et al. 2005).

Both non-oil economy and employment are particularly dependent on SMEs in Iran. SMEs can play a key role in driving the economy and employment if they are properly supported by the government (Saadatyar et al. 2020). In the recent decade, supporting programs for SMEs have been seriously pursued by the Iran Small Industries and Industrial Parks Organization (ISIPO). One of the most important programs covers the development of industrial clusters, and a significant portion of the total available budget is assigned to this program (Hassanpour and Pamucar 2019).

Given that there are many potential industrial clusters in different provinces in Iran, the government cannot simultaneously develop all of them due to financial constraints. Therefore, a certain number of higher priority clusters in each province are selected to be developed in collaboration with the private sector known as cluster development agents. Prioritization of industrial clusters in Iran is usually based on the outputs of provincial projects rather than practical scientific methods. In other words, a set of limited criteria is used in basic methods that cannot result in reliable outputs; therefore, operational plans of ISIPO could be adversely affected in the field of industrial clusters development.

Currently, the lack of appropriate scientific methods to prioritize industrial clusters is considered as a management problem in Iran. In the scientific literature there are few studies on the development of decision-making models for industrial clusters evaluation (Cantner et al. 2019). Moreover, the management of financial resources for the implementation of industrial cluster development plans (ICDPs) is also considered as a tactical decision-making problem since the limited resources of the government cannot fully cover all ICDPs (Zastupov 2019). It is better to make budget assignment based on the level of importance of each operational plan. Despite the importance of financial resources management, this problem also has not been quantitatively studied in the literature (Cao and Shi 2020).

The main purpose of this paper is to answer the question of how to make a decision-making model to prioritize potential industrial clusters and optimize the assignment of governmental funding to ICDPs. To this end, the development of SMEs in the form of industrial clusters is studied through a decision-making optimization framework in the South Khorasan province of Iran. The first phase of this framework includes a multiple criteria decision making (MCDM) model for evaluating industrial clusters based on criteria and sub-criteria extracted from upstream documents of ISIPO as well as national experts’ opinions. In this phase, global weights of criteria are determined using the best-worst method (BWM)(Rezaei 2015) and then prioritization of potential clusters is done using VIKOR (Opricovic 1998) so that the highest priority cluster can be selected for development in the province. In the second phase of the proposed framework, a robust multi-objective optimization model is developed to optimally assign government budget to ICDPs. The objective functions of this model include (1) maximization of economic growth, (2) maximization of social growth, and (3) minimization of implementation risk of cluster plans. Constraints make sure that the total funds assigned to action plans do not exceed the available budget, guarantee that the financial resources assigned to each plan are within predetermined limits, and ensure that only appropriate types of incentives are used for each action plan. As it is difficult to determine the true values of parameters related to economic growth, social growth, risk, and the importance of each plan, these are considered under interval uncertainty. The robust programming proposed by Bertsimas and Sim (2004) is applied to deal with this uncertainty.

The remaining part of the paper is as follows. Section 2 presents relevant literature from the field of industrial clusters management. Then the proposed MCDM approach, the mathematical model and uncertainty conditions are presented in Sect. 3. The solution method and case study are stated in Sect. 4 and Sect. 5, respectively. The obtained computational results of the MCDM phase and optimization phase as well as sensitivity analysis are reported in Sect. 6. The managerial insights are stated in Sect. 7, before concluding remarks are given in Sect. 8.

## Background and relevant literature

### Development of industrial clusters

The development of economic activities formed in specific geographical locations based on the natural potentials in the same region, is considered one of the most effective strategies to reach a sustainable economy (Lazzeretti et al. 2017). Existence of similar threats and opportunities, high social and cultural capitals, and institutionalization of technical skills in production are the most important features that can be observed in this type of economic activity (Munday et al. 2011). Since active enterprises in this field are usually small or medium-sized, the development of their activity in the industrial dimension is usually considered in the context of industrial clusters. This concept was first proposed by Marshall (1920) and is now considered by international organizations, including the United Nations Industrial Development Organization, as an attractive strategy for maintaining the benefits of industrial development in various products.

Some countries have executed special programs to improve activities of industrial clusters in the fields of energy, agriculture, service, and production (Toma et al. 2014). The policies for developing SMEs are often presented as a set of operational strategies with direct support of the government and the private sector. Some of the most important cluster policies include 1) broker policy, 2) demand-side policy, and 3) training policy (Barbieri et al. 2012). The government, being responsible for developing SMEs in the form of industrial clusters, implements a specific set of programs within each of these policies. In fact, government agencies have spent a significant amount of time and money on training, building infrastructure, improving production and sales, and developing logistics networks and supply chains in a limited period of time so that cluster members can be empowered and able to operate.

### Evaluation of industrial clusters

Financial and operational constraints have prevented governments from simultaneously implementing the plans for all potential industrial clusters (Spencer et al. 2010). Therefore, it is interesting for managers of governmental organizations to know how to identify high-priority industrial clusters to achieve the highest level of economic development and productivity. A set of criteria for evaluating sustainability of SMEs was presented by Malesios et al. (2020). A related set of criteria for proper evaluation in the context of industrial clusters is described below.

**Economic criterion** This criterion directly evaluates the economic activities of industrial clusters. Managers of governmental organizations tend to know which of the existing industrial clusters can strengthen the economic dimension in sustainable industrial development (Spencer et al. 2010). Hence, some helpful criteria include the cost of building a new firm in the cluster, the cost associated with training and development of cluster enterprises, the amount of added value created in cluster products, the level of cluster operators income, and the potential to increase the total revenue of the cluster.

**Social criterion** One of the main advantages of industrial development is the improvement social aspects (Basakha and Kamal 2019), including job creation. Employment has always been considered by researchers and managers of industrial development as one of the most common indicators of sustainable development (Asadullah and Savoia 2018). In addition, potential for brand development and the attractiveness of the products produced in the cluster for customers can effectively play a main role in the success of industrial cluster enterprises (Lai et al. 2005).

**Export criterion** The issues of international markets development and creation of international sales channels have always been among the most important goals of industry owners in various sectors. Export development criteria that can help improving the current situation of industrial clusters include competing in international markets, attracting global customers, and introducing cluster products in other countries (branding).

**Innovation and scalability criterion** The rapid advancement of technology and the unprecedented growth in international communications during globalization causes international competition to be directly related to innovation and scalability competition (Bresnahan and Gambardella 2004). One of the indicators of development is the research and development units and this factor causes the clear separation of rich countries from poor ones. One of the most important achievements of innovation and scalability is the commercialization of products worldwide. In economic markets, scalability means the extent to which enterprises can respond economically to increased demand. An expandable cluster is one that can increase its profits by increasing sales (Rocha 2004). In recent years, when access to modern technologies has become much easier, development efficiency has become much more important, and many industrial enterprises have been seeking to develop and expand themselves.

Applying the mentioned criteria enables a suitable evaluation of industrial clusters. In the following, some related studies to the field of research are reviewed.

There are different studies addressing economic aspects of industrial clustering. Kamran et al. (2017) focused on improving the domestic investment environment in different industrial clusters in Pakistan. Ahmed and Malik (2012) suggested to provide low-interest loans and also commercial budgets for SMEs in clusters. Götz (2020) suggested exploiting the cluster concept to attract investments for industries dealing with changes caused by fourth industrial revolution. Saadatyar et al. (2020) have recently studied the existing conditions of a carpet cluster in Iran. They observed export issues in the cluster caused by poor economic conditions. From the perspective of social development related to economic aspects (Poveda 2011), Rocha et al. (2020) emphasized the effect of clustering on economic and social integration within a geographical region by proposing a system dynamics model. They studied different factors related to employment growth, cohesion between regional workers, and new connections between enterprises. These factors have also been studied in the context of the US and Europe (Adam Cobb 2016; Konstantynova and Wilson 2017). Another important point is sharing social benefits between cluster members through formulating suitable policies (Asadullah and Savoia 2018; Garretsen et al. 2013). The social capital resulting from promoting cooperation among members is also a key factor of successful clusters (Wasiluk 2017). Another research direction is about a close connection between social development and the innovation level of the cluster (Lundvall et al. 2011), in particular for high-tech clusters (Rocha 2004).

Barbieri et al. (2020) considered the effective organizational, social, and economic criteria on innovation and sustainability of information and communication technologies clusters. In this regard, some researchers believe that special clusters, like high-tech production clusters, are more innovative due to their nature (Bresnahan and Gambardella 2004). On the other hand, some believe that the level of innovation mainly depends on the production technology in the cluster (Pietrobelli and Rabellotti 2011), or the educational level of the workers (Lai et al. 2005; Wei et al. 2007). Ai et al. (2020) examined the effect of networks on innovation and knowledge in China. For this purpose, they presented a conceptual model and worked on natural and intentional industrial clusters. Huang and Wang (2018) discussed a plastic industrial cluster with the aim of analyzing innovation networks and different factors related to them such as enterprise learning and network relations. Otsuka and Ali (2020) focused on converting agriculture clusters into agro-industrial clusters. They highlighted the role of managerial and technical training to increase innovation in the clusters as mentioned by Higuchi et al. (2019), Mano et al. (2012), and Sonobe and Otsuka (2010).

According to the literature, the development of industrial clusters has a significant effect on the local economy. Most studies in this field have mainly focused on product and market development strategies and on organizational management challenges. However, the evaluation of industrial clusters and the assignment of financial budgets to action plans are known as critical managerial decisions for decision makers. In addition, funding of action plans is one of the most important steps in the process of developing industrial clusters. This funding is usually provided by the public sector. Nonetheless, how to properly allocate funds to each action plan is considered as an important management decision that has not been explored in the research literature.

To investigate the existing gaps, this research develops a hybrid evaluation model based on MCDM methods and multi-objective optimization model. The most important novelties of this research include (1) extending a hybrid approach based on MCDM methods and multi-objective optimization to evaluate industrial clusters, (2) assigning government budget to each action plan optimally and (3) considering a comprehensive set of criteria and sub-criteria to evaluate industrial clusters.

## Research method

**Fig. 1:**
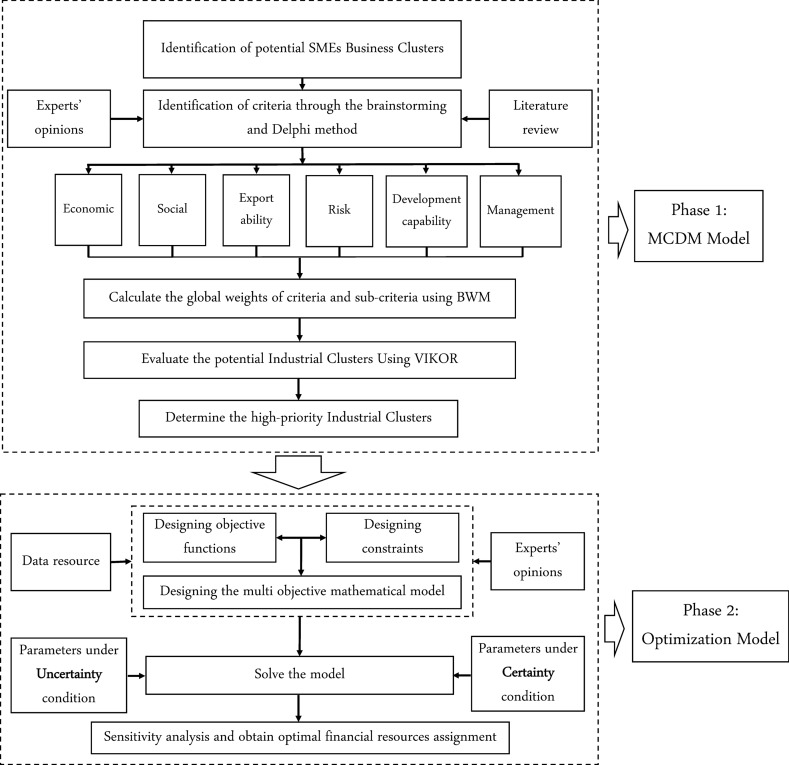
Flowchart of the proposed research method

The proposed framework consists of two phases. In the first phase, industrial clusters are evaluated using a MCDM model based on a set of criteria approved by SME development experts. In the second phase, the assignment of governmental funding to ICDPs is studied through a three-objective mathematical programming model under uncertainty with the goal of improving the implementation of development plans. Figure 1 shows a flowchart for the proposed research method. The output of this framework includes the priority of potential industrial clusters based on criteria and sub-criteria approved by experts, as well as the optimal assignment of governmental resources to development plans. Sensitivity analyses are carried out to evaluate the obtained results. In this way, the behavior of the proposed framework can be examined in different situations. Moreover, as the main beneficiaries of this research, industrial development managers can observe the effect of their decisions on the final results in different situations, and consequently they can make logical decisions based on political, environmental, and regional conditions of the desired cluster.

### Phase 1: The MCDM model

In this phase, the weights of criteria and the prioritization of industrial clusters are determined. A two-step procedure is applied to identify criteria and sub-criteria. In the first step, a wide range of criteria are extracted from upstream documents of ISIPO as well as from national experts’ opinions and the research literature. In the second step, as shown in Fig. 1, brainstorming and Delphi methods (Okoli and Pawlowski 2004) are used as an on/off method to screen the criteria.

**Fig. 2:**
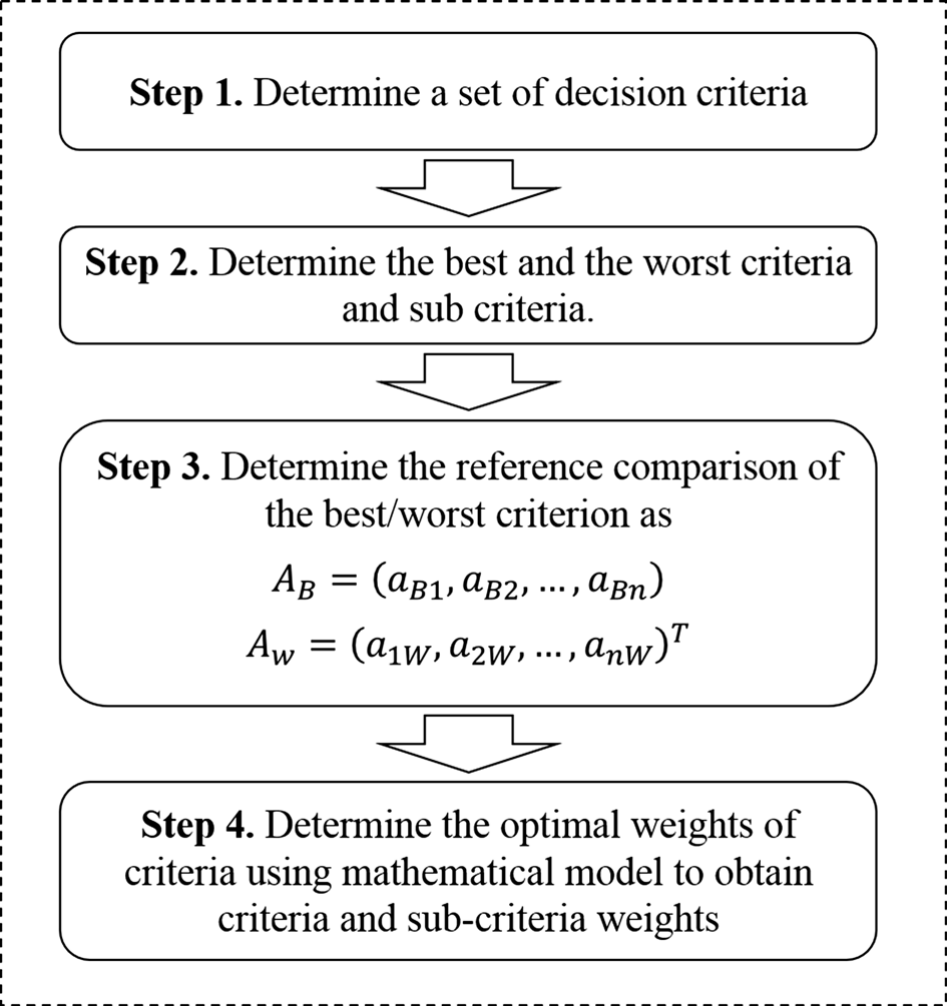
Flowchart for the best-worst method

Then, pairwise comparisons of BWM are performed to determine weights for each criterion and sub-criterion according to questionnaires completed by the experts. Optimal weights are obtained through a mathematical model with a low number of calculations (Rezaei 2016). Since the experts have different opinions about the best and the worst criteria, different final weights for each criterion are determined by each expert. A unique final weight for each criterion is based on taking a simple average of final weights (Rezaei 2015). Figure 2 shows the flowchart for BWM. Moreover, the steps of the BWM are described in detail in the supplementary material.

**Fig. 3:**
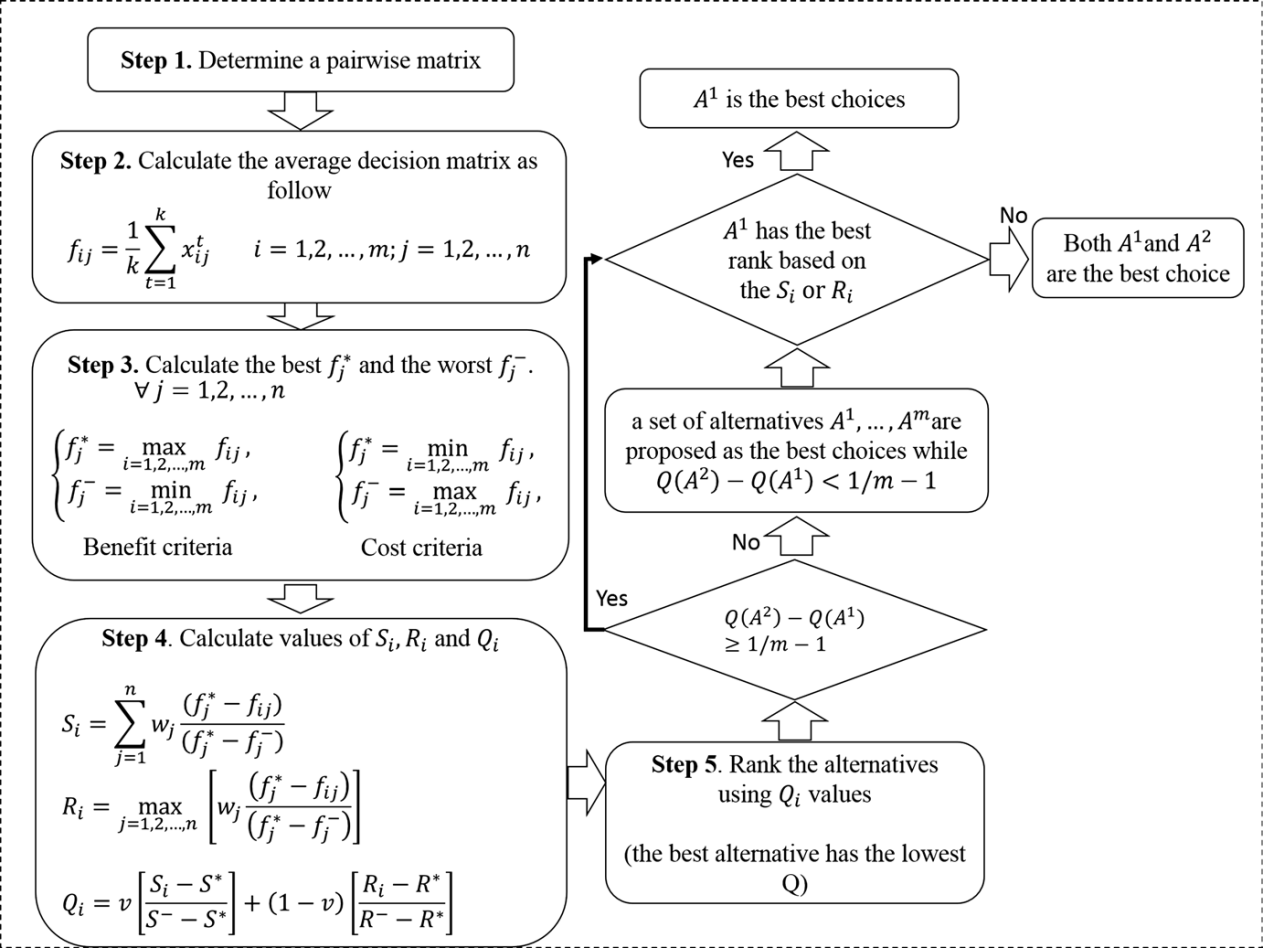
Flowchart for the VIKOR method

Finally, the priority of industrial clusters is determined using an option-criterion matrix of VIKOR as provided by experts. The power of VIKOR is to obtain the final ranks considering closeness to the ideal solution (Opricovic 1998). Figure 3 shows the flowchart for VIKOR and the steps of this method are described in detail in the supplementary material. In Fig. 3, *i* is the index of an option, *j* is the index of a criterion, *t* is the index of an expert, *m* is the number of options, and *n* is the number of criteria. The robustness of the choices made by VIKOR is then evaluated using a method used by Mir et al. (2021).

Although we chose to apply BWM and VIKOR in this work, other MCMD methods are available. To select among them, one alternative is to compare the obtained results from different methods (Kannan et al. 2021). The BWM is designed based on pairwise comparisons as the AHP. However, the number of pairwise comparisons of BWM is fewer than those of AHP. If there exist *n* decision criteria in a MCDM problem, BWM requires only $2n-3$ comparisons, compared to $n(n - 1)/2$ for AHP (Rezaei 2016). The final results of VIKOR represent a compromise in terms of distance to the ideal solution (Opricovic 1998).

### Phase 2: The multi-objective optimization model

The optimal assignment of funding to business development projects has always been a complex decision in the field of resource management, and requires the use of efficient methods (Rupeika-Apoga and Danovi 2015). Therefore, mathematical models can be used as an effective decision-making tool. The output of phase 1 includes the determination of the first-ranked cluster. In the second phase, a multi-objective mathematical model is used to optimally assign the financial resources of the government to the ICDPs. Generally, there are nine main action plans for developing industrial clusters in Iran, as shown in Table 1. Due to the limited financial resources of the government, proper planning is required to assign resources to each plan according to the opinions of experts and the cluster development agent, as well as the goals of the government. These goals mainly include maximizing economic growth, maximizing social growth (employment), and minimizing the risk of implementing action plans, yielding three corresponding objective functions in the proposed model.

**Table 1:** The main action plans for developing industrial clusters in Iran

No	Action plan
P1	Cluster law and policy studies
P2	Marketing and trade development
P3	Cluster management system development
P4	Investment and financing planning
P5	Cluster enterprises diagnosing
P6	Information technology development
P7	Production and technology development
P8	Infrastructures development
P9	Raw materials planning

Depending on the nature of action plans, the government can offer four types of incentives for implementing the plans: (1) providing enterprises with technical and human support, (2) reducing or eliminating asset taxes for enterprises (3) reducing or eliminating sales taxes for enterprises, and (4) providing cash payments. The type of government support is also determined by the proposed optimization model, which is presented next.

#### Indices

*i:* action plan, from the set $\{1,\ldots ,I\}$
*j:* government incentive type, from the set $\{1,\ldots ,J\}$

#### Input parameters

*E*_*ij*_: effect on cluster economic growth from implementing action plan *i* with incentive *j*
*S*_*ij*_: effect on cluster employment growth from implementing action plan *i* with incentive *j*
*R*_*ij*_: risk level from implementing action plan *i* with incentive *j*
*P*_*ij*_: equals 1 if action plan *i* can be implemented with incentive *j*, and 0 otherwise
*L*_*i*_: lower bound of budget assignment to action plan *i*
*U*_*i*_: upper bound of budget assignment to action plan *i*
*B*: total available budget

#### Decision variable

$$X_{ij}$$
: amount of financial support assigned to plan *i* using incentive type *j*

1 $$\begin{aligned} \max Z_1= & {} \sum _{i=1}^{I} \sum _{j = 1}^{J} E_{ij} X_{ij} \end{aligned}$$

2 $$\begin{aligned} \max Z_2= & {} \sum _{i=1}^{I} \sum _{j = 1}^{J} S_{ij} X_{ij} \end{aligned}$$

3 $$\begin{aligned} \min Z_3= & {} \sum _{i=1}^{I} \sum _{j = 1}^{J} R_{ij} X_{ij} \end{aligned}$$

subject to

4 $$\begin{aligned}{} & {} L_i \le \sum _{j=1}^{J} X_{ij} \le U_i,\quad i = 1, \ldots , I, \end{aligned}$$

5 $$\begin{aligned}{} & {} \sum _{i=1}^{I} \sum _{j = 1}^{J} X_{ij} \le B, \end{aligned}$$

6 $$\begin{aligned}{} & {} X_{ij} \le BP_{ij},\quad i = 1, \ldots , I, j = 1, \ldots , J, \end{aligned}$$

7 $$\begin{aligned}{} & {} X_{ij} \ge 0,\quad i = 1, \ldots , I, j = 1, \ldots , J. \end{aligned}$$

The first and the second objective functions are respectively to maximize economic growth and employment growth (job creation) of the cluster through implementation of each action plan. The third objective function minimizes the risk of implementing each plan. Constraints (4) guarantee that the assigned budget to each action plan meets pre-determined lower and upper bounds. Constraints (5) ensure that the amount of assigned financial incentives to action plans do not exceeded from available budget. Constraints (6) guarantee that only compatible incentive types are used to fund an action plan. Constraints (7) define the domains of the decision variables.

#### The mathematical model under uncertainty conditions

Since parameters $E_{ij}$, $S_{ij}$ and $R_{ij}$ are approximated based on opinions of experts and the cluster development agent, determining their true values is difficult. Robust programming is a technique that deals with uncertainty of parameters in a mathematical programming model (Bertsimas and Sim 2004). The method does not require a probability distribution over the uncertain parameters, and instead assumes that the true value of each parameter is located within an interval with given upper and lower bounds. This is commonly expressed in terms of a nominal value and a maximum and minimum deviation. In our case, we can formulate this in the form of expressions (8) to (10):

8 $$\begin{aligned}{} & {} \tilde{E}_{i,j} \in [E_{ij}-{\hat{E}}_{i,j},E_{ij}+{\hat{E}}_{i,j}] \end{aligned}$$

9 $$\begin{aligned}{} & {} \tilde{S}_{i,j} \in [S_{ij}-{\hat{S}}_{i,j},S_{ij}+{\hat{S}}_{i,j}] \end{aligned}$$

10 $$\begin{aligned}{} & {} \tilde{R}_{i,j} \in [R_{ij}-{\hat{R}}_{i,j},R_{ij}+{\hat{R}}_{i,j}] \end{aligned}$$

where $\tilde{E}_{i,j}$, $\tilde{S}_{i,j}$ and $\tilde{R}_{i,j}$ are the true values of the parameters. The nominal values of the parameters are ${E}_{i,j}$, ${S}_{i,j}$, and ${R}_{i,j}$, while ${\hat{E}}_{i,j}$, ${\hat{S}}_{i,j}$, ${\hat{R}}_{i,j}$ represent the maximum deviations from the nominal value for each parameter. Bertsimas and Sim (2004) proposed that it is unlikely for all the uncertain parameters to take on their worst possible value simultaneously. In robust programming, we therefore define an uncertainty budget, and we seek a solution that is best possible when at most a given number of uncertain parameter simultaneously take on their worst possible value.

Define $Y=\{1,\ldots ,I\}\times \{1,\ldots ,J\}$. Then, let parameters ${\Gamma _{1},\Gamma _{2},\Gamma _{3}}$ control the maximum number of parameters that can deviate from their nominal value in each of the three objective functions. When $\Gamma _{1} = \Gamma _{2} = \Gamma _{3} = 0$ there is no uncertainty in the problem, and when $\Gamma _{1} = \Gamma _{2} = \Gamma _{3} = |Y|$ all the parameters may take their worst case value at the same time (Soyster 1973). Thus, the most interesting solutions are found when $0< \Gamma _{1},\Gamma _{2},\Gamma _{3} < |Y|$. With this in mind, the robust version of the problem can be rewritten as follows.

11 $$\begin{aligned} \max \tilde{Z}_1= & {} \sum _{i=1}^{I} \sum _{j = 1}^{J} E_{ij} X_{ij} - \theta _1 \end{aligned}$$

12 $$\begin{aligned} \max \tilde{Z}_2= & {} \sum _{i=1}^{I} \sum _{j = 1}^{J} S_{ij} X_{ij} - \theta _2 \end{aligned}$$

13 $$\begin{aligned} \min \tilde{Z}_3= & {} \sum _{i=1}^{I} \sum _{j = 1}^{J} R_{ij} X_{ij} + \theta _3 \end{aligned}$$

subject to

14 $$\begin{aligned}&({4})-({7}), \nonumber \\ \theta _1&= \max _{\begin{array}{c} S:S\subseteq {Y}, |S| \le \Gamma _{1}, \\ (k,m) \in Y {\setminus } S \end{array} } \bigg (\sum _{(i,j) \in S} {\hat{E}}_{ij} X_{ij} + (\Gamma _{1} - \lfloor \Gamma _{1}\rfloor ) {\hat{E}}_{km} X_{km} \bigg ), \end{aligned}$$

15 $$\begin{aligned} \theta _2&= \max _{\begin{array}{c} S:S\subseteq {Y}, |S| \le \Gamma _{2}, \\ (k,m) \in Y {\setminus } S \end{array} } \bigg (\sum _{(i,j) \in S} {\hat{S}}_{ij} X_{ij} + (\Gamma _{2} - \lfloor \Gamma _{2}\rfloor ) {\hat{S}}_{km} X_{km} \bigg ), \end{aligned}$$

16 $$\begin{aligned} \theta _3&= \max _{\begin{array}{c} S:S\subseteq {Y}, |S| \le \Gamma _{3}, \\ (k,m) \in Y {\setminus } S \end{array} } \bigg (\sum _{(i,j) \in S} {\hat{R}}_{ij} X_{ij} + (\Gamma _{3} - \lfloor \Gamma _{3}\rfloor ) {\hat{R}}_{km} X_{km} \bigg ). \end{aligned}$$

Equations (14) to (16) make the problem nonlinear and to efficiently solve the problem they should be linearized. This can be achieved by following the method of Bertsimas and Sim (2004). We illustrate this method on equation (14) and objective function (11), and note that $\theta _2$ and $\theta _3$ can be handled in the same way. For given values of $(X_{ij})_{i={1,\ldots ,I},j={1,\ldots ,J}}$, we can linearize $\theta _1$ by first defining auxiliary variables $\lambda _{ij}^{E}$ with domains $0 \le \lambda _{ij}^{E} \le 1$. This leads to the following formulation for $\theta _1$:

17 $$\begin{aligned}&\theta _1 = \max \sum _{i=1}^{I} \sum _{j = 1}^{J} {\hat{E}}_{ij} X_{ij} \lambda _{ij}^{E} \end{aligned}$$

subject to

18 $$\begin{aligned}{} & {} \sum _{i=1}^{I} \sum _{j=1}^{J} \lambda _{ij}^{E} \le \Gamma _{1}, \end{aligned}$$

19 $$\begin{aligned}{} & {} \lambda _{ij}^{E} \le 1, \quad i = 1, \ldots , I, j = 1, \ldots , J, \end{aligned}$$

20 $$\begin{aligned}{} & {} \lambda _{ij}^{E} \ge 0, \quad i = 1, \ldots , I, j = 1, \ldots , J. \end{aligned}$$

The optimal solution for this formulation must have $\lfloor \Gamma _1 \rfloor$ variables $\lambda _{ij}^{E}=1$ and one variable $\lambda _{ij}^{E}=\Gamma _1 - \lfloor \Gamma _1 \rfloor$, with an optimal objective function value of $\theta _1$. To use this equivalence in linearizing the robust problem, strong duality is first exploited. Introduce a dual variable $\alpha ^E$ for constraint (18) and dual variables $\beta ^E_{ij}$ for constraints (19). Then, we can also find $\theta _1$ by solving the dual problem:

21 $$\begin{aligned}{} & {} \theta _1 = \min ~\Gamma _{1} \alpha ^{E} + \sum _{i = 1}^{I} \sum _{j = 1}^{J} \beta _{ij}^{E} \end{aligned}$$

subject to

22 $$\begin{aligned}{} & {} \alpha ^{E} + \beta _{ij}^{E} \ge {\hat{E}}_{ij} X_{ij},\quad i = 1, \ldots , I, j = 1, \ldots , J, \end{aligned}$$

23 $$\begin{aligned}{} & {} \beta _{ij}^{E} \ge 0,\quad i = 1, \ldots , I, j = 1, \ldots , J, \end{aligned}$$

24 $$\begin{aligned}{} & {} \alpha ^{E} \ge 0. \end{aligned}$$

Now, by strong duality, any values of $\alpha ^E$ and $\beta ^E_{ij}$ that satisfy (22)-(24) give a value $\Gamma _{1} \alpha ^{E} + \sum _{i = 1}^{I} \sum _{j = 1}^{J} \beta _{ij}^{E} \ge \theta _1$, with the optimal values of $\alpha ^E$ and $\beta ^E_{ij}$ providing a value of exactly $\theta _1$. After performing similar conversions for each of the three objective functions, the robust problem can be formulated as a linear programming problem, where the expressions for $\theta$ are replaced by a corresponding expression consisting of the objective function in the above dual problem:

25 $$\begin{aligned}{} & {} \max \tilde{Z}_1 = \sum _{i=1}^{I} \sum _{j = 1}^{J} E_{ij} X_{ij} - \Gamma _{1} \alpha ^{E} - \sum _{i=1}^{I} \sum _{j = 1}^{J} \beta _{ij}^{E} \end{aligned}$$

26 $$\begin{aligned}{} & {} \max \tilde{Z}_2 = \sum _{i=1}^{I} \sum _{j = 1}^{J} S_{ij} X_{ij} - \Gamma _{2} \alpha ^{S} - \sum _{i=1}^{I} \sum _{j = 1}^{J} \beta _{ij}^{S} \end{aligned}$$

27 $$\begin{aligned}{} & {} \min \tilde{Z}_3 = \sum _{i=1}^{I} \sum _{j = 1}^{J} R_{ij} X_{ij} + \Gamma _{3} \alpha ^{R} + \sum _{i=1}^{I} \sum _{j = 1}^{J} \beta _{ij}^{R} \end{aligned}$$

subject to

28 $$\begin{aligned}&({4})-({7}),&~({22})-({24}), \nonumber \\&\alpha ^{S} + \beta _{ij}^{S}&\ge {\hat{S}}_{ij} X_{ij},&i = 1, \ldots , I, j = 1, \ldots , J, \end{aligned}$$

29 $$\begin{aligned}&\beta _{ij}^{S}&\ge 0,&i = 1, \ldots , I, j = 1, \ldots , J, \end{aligned}$$

30 $$\begin{aligned}&\alpha ^{S}&\ge 0, \end{aligned}$$

31 $$\begin{aligned}&\alpha ^{R} + \beta _{ij}^{R}&\ge {\hat{R}}_{ij} X_{ij},&i = 1, \ldots , I, j = 1, \ldots , J, \end{aligned}$$

32 $$\begin{aligned}&\beta _{ij}^{R}&\ge 0,&i = 1, \ldots , I, j = 1, \ldots , J, \end{aligned}$$

33 $$\begin{aligned}&\alpha ^{R}&\ge 0. \end{aligned}$$

## Solution method

**Fig. 4:**
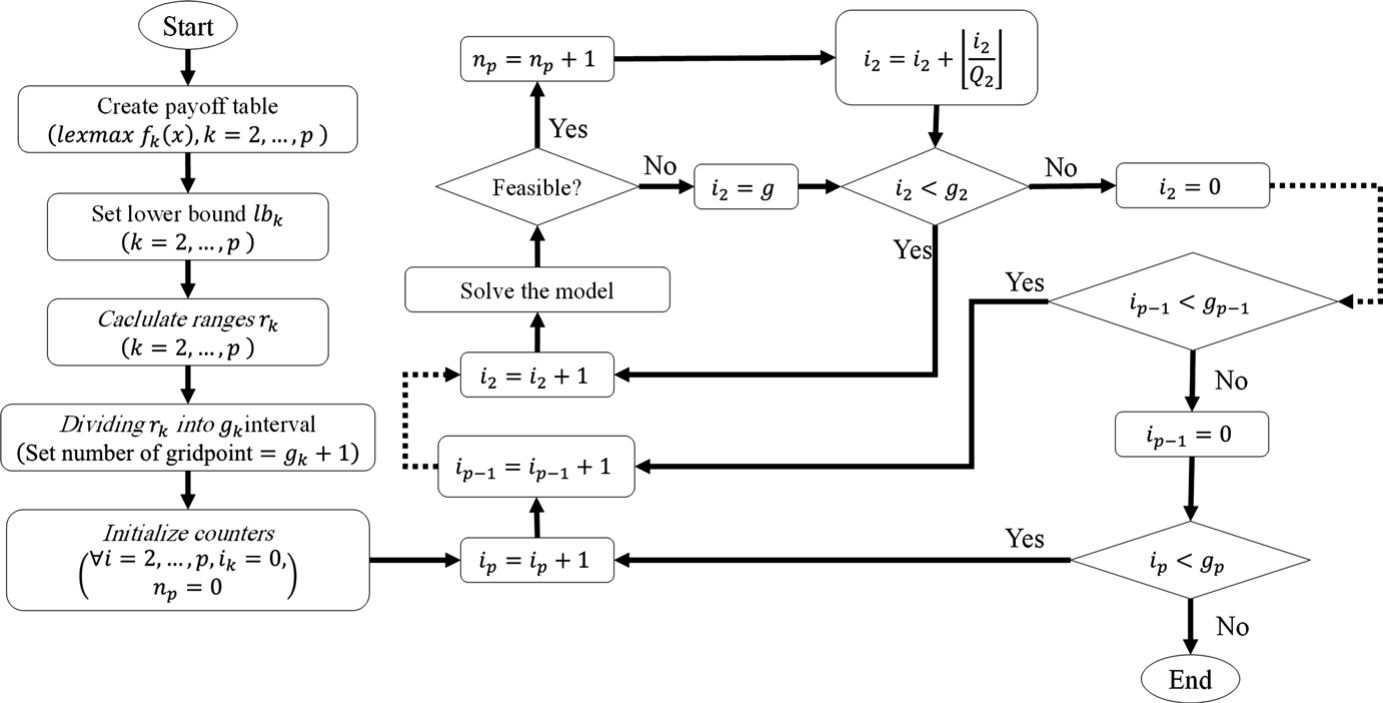
Flowchart of augmented epsilon-constraint method

In a multi-objective problem, there is not a single optimal solution, but rather a set of solutions forming a Pareto front. To obtain the optimal Pareto front, different methods have been proposed, out of which the augmented epsilon-constraint method has been found to be an efficient method (Mavrotas and Florios 2013). Let the general structure of a multi objective model be given as

34 $$\begin{aligned}&\max \bigg (f_{1}(x),f_{2}(x),\ldots ,f_{p}(x)\bigg ) \end{aligned}$$

subject to

$$\begin{aligned}&x \in S, \end{aligned}$$

where *x* is decision variable vector, $f_{1}(x),f_{2}(x),\ldots ,f_{p}(x)$ are objective functions, and *S* is the feasible space. In the augmented epsilon-constraint method, model (34) is converted to model (35) to achieve optimal Pareto front solutions:

35 $$\begin{aligned}&\max \bigg (f_{1}(x)+\epsilon \big (\frac{s_{2}}{r_{2}} + 10^{-1} \times \frac{s_{3}}{r_{3}} +\cdots + 10^{-(p-2)}\times \frac{s_{p}}{r_{p}} \big ) \bigg ) \end{aligned}$$

subject to

$$\begin{aligned}&f_{i}(x) - s_{i} = e_{i},&i = 2, \ldots , p,\\&x \in S, \\&s_{i} \ge 0,&i = 1, \ldots , p,\\ \end{aligned}$$

where $e_{2},e_{3},\ldots ,e_{p}$ are right hand side values for a specific iteration of the method, $r_{2},r_{3},\ldots ,r_{p}$ are the ranges of the objective functions, $s_{2},s_{3},\ldots ,s_{p}$ are auxiliary variables of constraints, and epsilon is a small number. Figure 4 shows the steps of implementing the augmented epsilon-constraint method (Mavrotas and Florios 2013).

In the figure, $e_{k}=l_{k} + i_{k} Q_{k}$, $g_{k}$ is the number of intervals for objective function *k*, $l_{k}$ is the lower bound for objective function *k*, $Q_{k}=r_{k}/g_{k}$ is the step for the objective function *k*, and $n_{p}$ is number of Pareto optimal solutions to be found.

## Case study

ISIPO has the main responsibility for developing industrial clusters in Iran. This organization implements the development plans of industrial clusters in three-year periods with the help of cluster development agents. As several potential new clusters can be identified in Iran, ISIPO managers and cluster development agents select the high-ranked industrial clusters and create financial plans for developing them. The decision makers in this process are ISIPO managers, who can use the results of our analysis to improve management decisions. Employees working in the small industries department in ISIPO as well as cluster development agents are considered as experts and have provided inputs to the following case study.

With the aim of identifying opportunities for developing SMEs in the South Khorasan province, ten industrial clusters are identified as shown in Table  2. Each cluster can be developed at regional, national, and international levels, leading to social and economic growth in the province. Cluster development projects are implemented by cluster development agents supported by governmental organizations in Iran, similar to what takes place in pioneering countries in the field of SME development. As shown in Table 1, there are nine main action plans for developing industrial clusters in Iran. The implementation of action plans directly depends on the assigned governmental budget. Managers of ISIPO in South Khorasan want to apply a comprehensive and effective method to select a cluster with the highest priority. Moreover, they want to simultaneously manage financial resources to implement the desired action plans proposed by the cluster development agent, because they aim to reach the maximum level of economic and social growth.

**Fig. 5:**
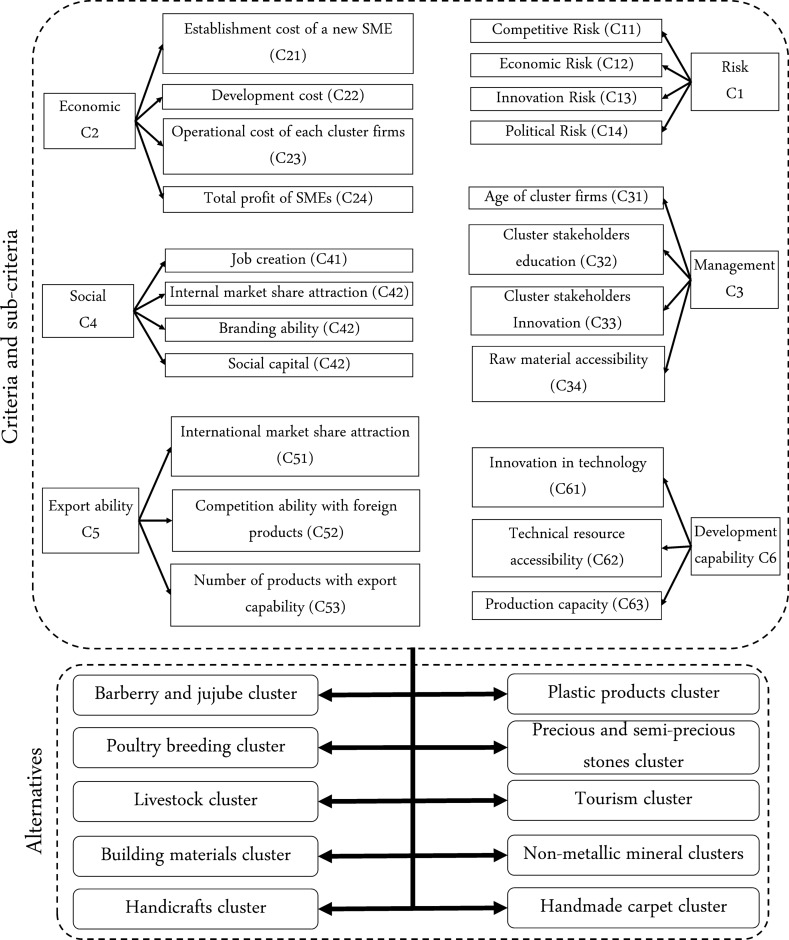
The conceptual model in the MCDM phase

To solve the problem, the proposed framework is presented to the managers as a managerial tool. Effective criteria and sub-criteria in evaluating industrial clusters are identified through face-to-face interviews with members of the SME development committee in ISIPO. Figure 5 shows the related conceptual model. The figure contains three economic sub-criteria. When it comes to ICDPs, it is important to differentiate between the establishment cost, the development cost, and the operational cost of an SME. The cost of establishing an SME comprises the investments to register and to start the activity of the enterprise. On the other hand, the development costs mainly include product line updates, manpower training, and programs for research and development that are executed periodically over a limited period of time. Finally, the operational cost contains elements such as personnel salaries, raw material purchase cost, and logistics and transportation costs.

**Table 2:** The identified industrial clusters in South Khorasan province, Iran

ID	Description
A1	Tourism
A2	Precious and semi-precious stones
A3	Non-metallic minerals
A4	Concrete materials
A5	Handmade carpet
A6	Barberry and jujube
A7	Plastic products
A8	Livestock
A9	Handicrafts
A10	Poultry farming

Considering that the proposed approach of this research combines models of MCDM and multi-objective optimization, this section describes the input data required for each of the models. Generally, the input data of the MCDM phase includes the scores related to the pairwise comparison between the criteria and sub-criteria based on the opinions of experts, which are collected through a questionnaire. For this purpose, the questionnaire based on the pattern of BWM is designed and provided to experts from the private sector and also the ISIPO managers in the province. After implementing the MCDM model, a meeting was held with the presence of the mentioned individuals to analyze the numerical results and the final selection of the industrial cluster. The input data required to run the mathematical model includes the output of the MCDM phase for selecting the highest priority industrial cluster for development as well as the numerical values of the parameters presented in Sect. 3.2. The ISIPO annual financial documents were used to collect the data required for the mathematical model.

Input data is required for the MCDM model in the first phase and the multi-objective optimization model in the second phase. In the first phase, a questionnaire based on a linguistic scale from one to nine is used for BWM and a questionnaire based on a linguistic scale from one to five is designed for VIKOR. In the BWM questionnaire, pairwise comparisons are made between the criteria to select the best and the worst criteria, rate the best criterion over the other ones, and rate other criteria over the worst one. The VIKOR questionnaire involves determining the importance of each sub-criterion.

The parameters for the multi-objective optimization model are determined based on the opinions of senior managers in combination with the databases of ISIPO in South Khorasan. The total available budget and the upper and lower limits of the budget allocated to each plan are determined based on national guidelines approved by the Ministry of Industry, Mines and Trade in Iran. One important point in solving the optimization model is to determine the coefficients of the objective functions. Some criteria related to determining the coefficients of the objective functions are qualitative and determined based on the opinions of experts within the organization. For example, to determine the economic growth rate of the cluster for the implementation of each action plan, a council consisting of consultants and experts is created and then the impact of the implementation of each action plan on the criteria affecting the economic growth of clusters is estimated. The coefficients of the objective functions are thus determined based on the studied conditions, historical data, as well as expert opinion.

## Computational results

In this section, results of the MCDM phase and the multi-objective optimization model are investigated. First, weights of criteria and sub-criteria are determined by BWM implemented using CPLEX (ILOG 2018). Then prioritization results of industrial clusters are obtained using VIKOR coded in MATLAB. Finally, the multi-objective optimization model is implemented using CPLEX and the efficiency of the proposed model for optimal assignment of governmental budgets is examined.

### The computational results of the MCDM model

Ten industrial clusters are identified to develop SMEs in South Khorasan province as listed in Table  2. These industrial clusters are prioritized by considering six groups of criteria including risk criteria, economic criteria, management criteria, social criteria, development criteria, and export criteria. The experts working in ISIPO were asked to complete pairwise comparisons, and Table 3 shows final weights of the criteria. All calculations related to determining the criteria and sub-criteria weights are provided in the supplementary material.

**Table 3:** Final weights of effective factors in evaluating industrial clusters obtained by BWM

Criterion	Weight	Rank	Sub-criterion	Code	Local weight	Global weight	Rank
Risk	0.073	6	Competitive Risk	$C_{11}$	0.226	0.017	19
			Economic Risk	$C_{12}$	0.428	0.031	12
			Innovation Risk	$C_{13}$	0.105	0.008	21
			Political Risk	$C_{14}$	0.242	0.018	18
Economic	0.245	1	Establishment cost of a new SME	$C_{21}$	0.159	0.039	9
			Development cost of a new SME	$C_{22}$	0.334	0.082	5
			Operational cost of a new SME	$C_{23}$	0.131	0.032	11
			Total profit of SMEs	$C_{24}$	0.375	0.092	3
Management	0.079	5	Age of cluster	$C_{31}$	0.082	0.006	22
			Education of cluster stakeholders	$C_{32}$	0.144	0.011	20
			Innovation of cluster stakeholders	$C_{33}$	0.394	0.031	13
			Raw material accessibility	$C_{34}$	0.381	0.030	14
Social	0.211	2	Job creation	$C_{41}$	0.449	0.095	2
			Internal market share attraction	$C_{42}$	0.152	0.032	10
			Branding ability	$C_{43}$	0.282	0.060	8
			Social capital	$C_{44}$	0.116	0.025	16
Export	0.210	3	International market share attraction	$C_{51}$	0.543	0.114	19
			Competition ability with foreign products	$C_{52}$	0.344	0.072	6
			products with export capability	$C_{53}$	0.114	0.024	17
Development	0.182	4	Innovation in technology	$C_{61}$	0.455	0.083	4
			Technical resource accessibility	$C_{62}$	0.428	0.029	15
			Production capacity	$C_{63}$	0.383	0.070	7

Table 3 shows that international market share attraction, with a global weight of 0.114, is the most important criterion, while the age of the cluster, with a global weight of 0.006, is the least important. Furthermore, job creation, total profit of SMEs, innovation in technology, and development cost are also ranked as important criteria in evaluating the industrial clusters. Using the global weights and the criterion-decision matrix in Table 4, the final prioritization of industrial clusters is obtained by VIKOR. The compromise solution of VIKOR is stable within a decision-making process, which could be the strategy of maximum group utility (when $v > 0.5$ is needed), or by consensus $(v \approx 0.5)$, or with veto $(v < 0.5)$. Here, *v* is the weight of decision-making strategy of maximum group utility that is set to 0.5 in this paper.

**Table 4:** The criterion-decision matrix for prioritization of industrial clusters

Sub-criterion	Tourism	Precious and semi- precious stones	Non-metallic mineral	Building materials	Handmade carpet	Barberry and jujube	Plastic products	Livestock	Handicrafts	Poultry Breeding
Competitive Risk	3.29	2.57	3.00	3.43	3.29	4.29	2.57	3.43	3.86	2.71
Economic Risk	3.00	3.00	3.29	3.00	3.29	3.57	3.71	3.00	2.57	3.00
Innovation Risk	3.29	2.86	3.29	2.86	2.86	3.86	3.14	3.14	3.14	2.71
Political Risk	3.29	3.57	3.29	2.71	2.29	3.57	3.00	2.71	3.29	2.71
Establishment cost of a new SME	2.86	2.71	3.14	3.14	2.86	4.00	3.86	3.00	2.57	3.29
Development cost	2.57	2.71	2.86	2.57	2.71	4.29	2.86	2.14	2.57	3.14
Operational cost of each cluster firms	3.29	3.14	3.43	3.43	3.43	3.57	2.86	2.57	2.14	3.29
Total profit of SMEs	2.57	2.86	3.00	3.14	3.00	4.00	2.86	2.29	3.29	3.14
Age of cluster firms	2.86	3.14	3.29	2.86	3.43	4.14	3.14	2.86	3.43	3.00
Education of cluster stakeholders	2.86	3.14	3.00	2.57	3.14	4.43	3.00	3.00	3.00	2.43
Innovation of cluster stakeholders	3.00	2.86	2.43	3.29	2.71	4.14	3.29	2.43	2.57	2.57
Raw material accessibility	2.71	3.43	3.29	2.57	3.00	4.14	2.57	3.29	3.14	2.86
Job creation	3.29	2.57	3.00	2.57	2.86	4.14	3.00	3.00	3.00	2.71
Internal market share attraction	3.14	2.71	2.57	3.29	3.57	3.86	3.00	2.86	3.00	2.86
Branding ability	2.71	3.00	3.14	2.86	2.57	4.00	2.57	2.57	2.86	2.71
Social capital	3.00	3.43	2.86	3.14	3.43	4.14	2.71	2.57	3.14	3.14
International market share attraction	2.86	2.86	2.71	2.86	3.14	4.00	2.71	2.71	3.29	2.86
Competition ability with foreign products	2.71	2.71	3.14	3.57	3.29	3.71	2.86	3.00	3.00	3.14
products with export capability	3.29	2.71	2.57	2.86	2.86	4.14	3.00	3.14	3.14	2.71
Innovation in technology	3.14	3.00	2.86	2.86	2.57	3.43	3.00	2.71	2.86	3.43
Technical resource accessibility	3.14	2.57	3.43	2.86	3.29	4.14	2.57	2.57	2.71	3.43
Production capacity	2.86	3.14	3.00	2.86	3.14	4.00	2.71	2.71	2.43	3.14

Table 5 shows final ranks of industrial clusters. The cluster for barberry and jujube, with a score of 0.1364, takes the highest priority, while the cluster for plastic products, with a score of 0.996, has the lowest priority for development. Complete calculations of VIKOR are presented in the supplementary material file.

**Table 5:** The final prioritization of industrial clusters obtained by VIKOR

Rank	Q	Industrial Cluster
1	0.136	Barberry and jujube
2	0.346	Handicrafts
3	0.539	Handmade carpet
4	0.731	Poultry Breeding
5	0.745	Building materials
6	0.767	Tourism
7	0.784	Precious and semi-precious stones
8	0.941	Non-metallic mineral
9	0.983	Livestock
10	0.996	Plastic products

**Fig. 6:**
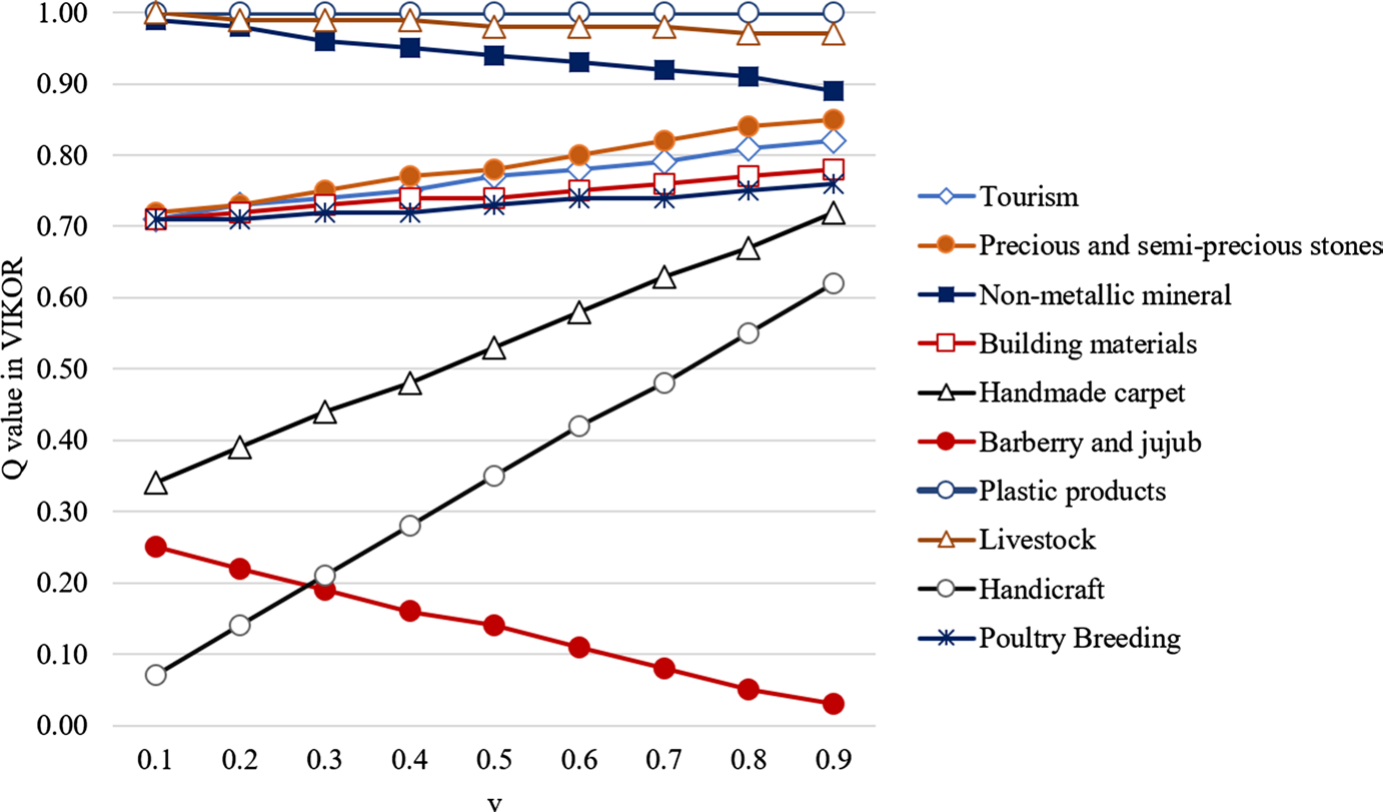
The effect of changing parameter *v* in clusters prioritization

Since the parameter *v* in VIKOR can be varied in the range 0 to 1, it can cause changes in the prioritization of alternatives. Analyzing these changes can help decision makers to choose the right value for the parameter. Figure 6 illustrates prioritization of the industrial clusters for different values of parameter *v*.

For $v=0.1$ and $v=0.2$ the best ranked cluster is handicrafts, with the second best being the cluster for barberry and jujube. However, for $v=0.3$ to $v=0.9$ these two clusters change places. Ranks of other cluster are stable across all values of the parameter *v*. This issue shows that the priority of developing the handicrafts cluster is close to that of barberry and jujube cluster; therefore, choosing the final cluster to be developed is a critical decision. The robustness of the VIKOR results is analyzed by varying the weight of each sub-criterion (Table 3) by $-15\%$, $-10\%$, $-5\%$, $+5\%$, $+10\%$, and $+15\%$. In all cases, when using $v=0.5$, the barberry and jujube cluster is found to be the first ranked cluster. Finally, the managers of ISIPO in South Khorasan selected the barberry and jujube cluster as the first-ranked cluster and decided to implement the development plans for this cluster.

### Results of mathematical model

After selecting the barberry and jujube cluster as the highest priority through implementing the hybrid MCDM model, financial incentives are assigned to action plans of the cluster. Table 6 presents parameters related to economic and social growth and risks of executing action plans. The action plans can be implemented in four different ways, including offering human and technological support (M1), reducing or eliminating asset tax (M2), reducing or eliminating sales tax (M3), and providing cash payments (M4). Only the objective function coefficients for action plans *i* and incentive types *j* where $P_{ij}=1$ are shown, while combinations where $P_{ij}=0$ are indicated by a hyphen. The parameters related to the objective functions are obtained as the average of experts ratings in percentages. Table 7 shows the available budget and lower and upper bounds of assigning financial incentives to each of the action plans from Table 1. Based on interviews with managers of ISIPO, the maximum available budget for implementing action plans is 71,000 dollar.

**Table 6:** Objective function coefficients for combinations of action plans *i* (P1-P9) and incentive types *j* (M1-M4)

		P1	P2	P3	P4	P5	P6	P7	P8	P9
$E_{ij}$	M1	0.11	0.79	–	0.40	0.72	0.17	0.16	0.39	0.80
	M2	0.56	0.65	0.29	–	0.38	0.40	0.76	0.26	0.75
	M3	0.23	0.17	0.54	0.76	0.14	–	0.21	0.14	0.34
	M4	–	0.10	0.44	0.65	–	0.47	–	0.34	–
$S_{ij}$	M1	0.41	0.53	–	0.26	0.13	0.48	0.43	0.62	0.71
	M2	0.20	0.31	0.77	–	0.48	0.70	0.52	0.51	0.18
	M3	0.68	0.25	0.16	0.34	0.27	–	0.17	0.75	0.28
	M4	–	0.23	0.15	0.59	–	0.69	–	0.8	–
$R_{ij}$	M1	0.40	0.69	–	0.33	0.52	0.38	0.70	0.78	0.51
	M2	0.39	0.76	0.61	–	0.17	0.13	0.44	0.47	0.44
	M3	0.23	0.54	0.42	0.35	0.75	–	0.51	0.43	0.63
	M4	–	0.64	0.78	0.12	–	0.52	–	0.54	–

**Table 7:** The minimum and maximum amounts of assigned budget to each action plan

USD	P1	P2	P3	P4	P5	P6	P7	P8	P9
$L_{i}$	778	679	410	616	645	753	563	569	577
$U_{i}$	9721	10,000	10,000	10,000	9672	10,000	8895	10,000	9137

**Fig. 7:**
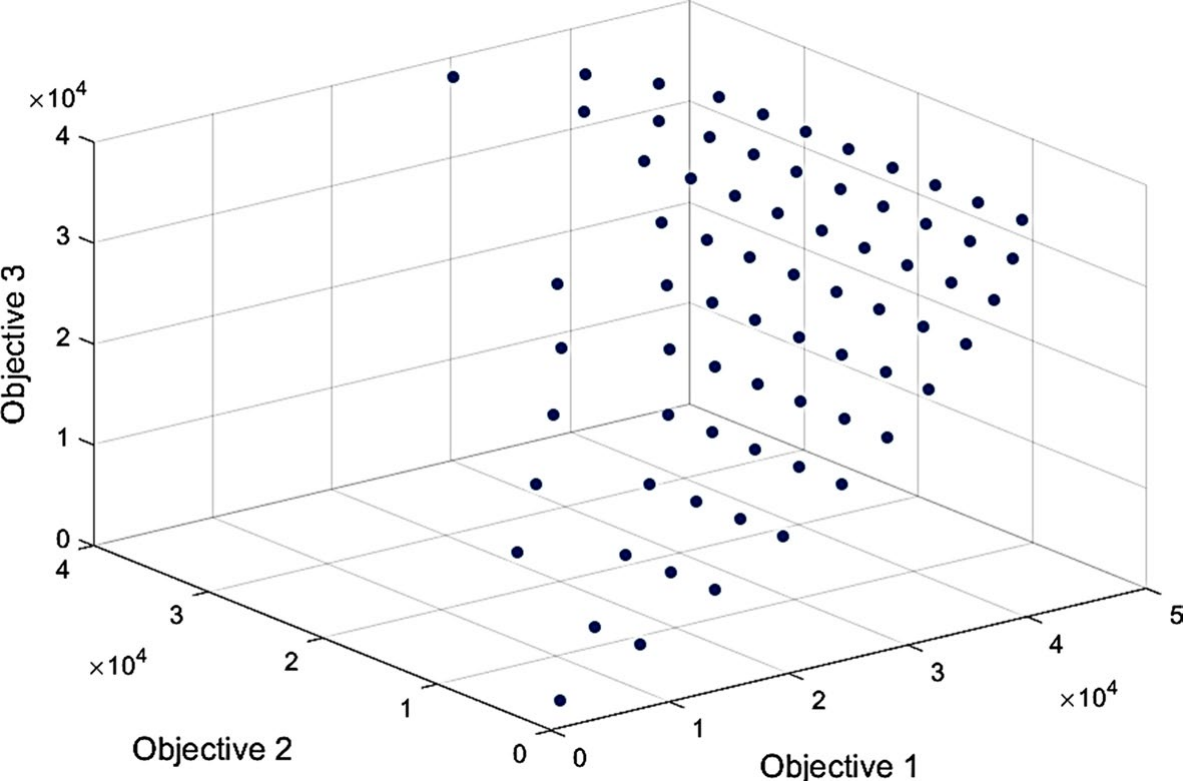
The obtained Pareto front

Managers of the cluster development department believe that the appropriate values for robustness parameters are $\Gamma _{1}=5$, $\Gamma _{2}=8$ and $\Gamma _{3}=10$. Figure 7 illustrates the Pareto front resulting from the proposed optimization model. The obtained Pareto front has 71 solutions ranging from 2,228.7 to 40,981.6 for the first objective function, 1,547.3 to 39,333.5 for the second objective function, and 1,657.1 to 38,413.4 for the third objective function. Moreover, the extreme (corner) solutions are (40,981.6, 1,547.3, 38,413.4), (29,564, 39,333.5, 38,413.4) and (2,228.7, 1,547.3, 1,657.1), in which each of the objective functions has its best value.

One of the most important challenges in solving multi-objective problems is choosing one of the efficient solutions in the Pareto-front to implement in real world conditions, since the Pareto-optimal solutions are non-dominated and have no priority over each other. To this end, a method is presented for calculating the efficiency level of each solution in the Pareto front based on its proximity to the ideal solution (the solution in which all objective functions have the best value).

### Selection of the most efficient Pareto solution

To choose a solution from the Pareto front, first the mathematical model is solved for each objective function and the optimal value of the objective functions is calculated separately. Then the Euclidean distance of each Pareto-optimal solution to the ideal solution is calculated. The solution with the shortest distance to the ideal solution is selected for implementation. The steps of this method are discussed as follows.

**Step 1:** Solve the mathematical model for each objective function separately and store the optimal values in $Z_{1}^{*}$, $Z_{2}^{*}$ and $Z_{3}^{*}$.

**Step 2:** Solve the mathematical model using the augmented epsilon-constraint method and store objective function values for the solutions in the set $S^{*}$.

**Step 3:** Calculate the Euclidean distance from solutions in the set $S^{*}$ to the ideal point $(Z_{1}^{*},Z_{2}^{*},Z_{3}^{*})$ based on Equation (36) and produce mean ideal distance (MID) values.

**Step 4:** Select the Pareto-optimal solution with the lowest MID value as the final solution.

The equation for calculating MID is

36 $$\begin{aligned}&MID_{i} = \sqrt{\sum _{j=1}^{p}(Z_{j}^{*} - Z_{ij})^2} ,&i \in S^{*}, \end{aligned}$$

where *p* is the number of objective functions, and $Z_{ij}$ is the value of the *j*th objective function for the *i*th solution in the Pareto front. After doing the related calculations, values of the first $(Z_{1}^{MID})$, the second $(Z_{2}^{MID})$, and the third $(Z_{3}^{MID})$ objective functions are 32,999.95, 24,219.03, and 23,710.87 for the best Pareto solution with $MID = 27,902.02$. For this solution, the optimal structure of the budget assigned to each action plan is shown in Table 8.

**Table 8:** The optimal assignment of governmental budget to each action plan

	P1	P2	P3	P4	P5	P6	P7	P8	P9	Sum
M1							6372			6372
M2	5086		2408		2203	8261			8500	26,458
M3	2882	9996						8319		21,197
M4			6749	9982						16,731
Sum	7968	9996	9157	9982	2203	8261	6372	8319	8500	70,758

Table 8 shows that a total of 70,758 dollar is spent, which is slightly less than the budget of 71,000 dollar expressed in constraint (5). Among the action plans, both marketing and trade development (P2) and investment and financing planning (P4) receive a 14% share of the total budget. Moreover, 13% of the total budget is assigned to cluster management system development (P3). Other action plans including information technology development (P6), infrastructures development (P8) and raw materials planning (P9) separately receive 12% of the total budget. The even division of financial resources indicates that the different action plans are of a similar importance. Interestingly, only 24% of government incentives is in the form of providing cash payments (M4), and 37% of the incentives is spent reducing or eliminating asset tax for enterprises (M2) which demonstrates the challenges faced by SMEs with respect to tax payments. Similarly, 30% of the incentives is related to reducing or eliminating sales tax of enterprises (M3) indicating management problems and poor communication between the SMEs and the tax system. Finally, offering enterprises technical and human support (M1) constitutes 9% of the total incentives.

### Sensitivity analysis

To investigate the sensitivity of the proposed model to uncertain parameters, different combinations of the robustness parameters are considered and the solution from the Pareto front with the best MID value for each combination is determined. Numerical comparisons are then provided to illustrate managerial perspectives. For this purpose, Table 9 shows computational results where $\Gamma _1$, $\Gamma _2$, and $\Gamma _3$ are varied from 5 to 34.

**Table 9:** Sensitivity analysis of the mathematical model over robustness parameters

Instance	$\Gamma _{1}$	$\Gamma _{2}$	$\Gamma _{3}$	$\min _{i \in S^{*}} MID_{i}$	$Z_{1}^{MID}$	$Z_{2}^{MID}$	$Z_{3}^{MID}$
1	5	20	34	24,565.90	40,224.20	1547.28	38,413.38
2	8	18	32	27,855.01	34,194.63	5325.91	38,413.38
3	10	16	30	29,365.05	29,724.21	9104.53	38,413.38
4	12	14	28	23,342.55	25,910.92	12,883.16	38,413.38
5	14	12	26	24,493.53	20,970.57	16,661.78	38,413.38
6	16	10	24	28,093.24	15,220.36	20,440.41	38,413.38
7	18	8	22	27,962.52	11,768.03	24,219.03	34,737.75
8	20	5	20	28,615.19	9591.14	27,997.65	31,062.12
9	22	34	18	28,221.25	9020.72	31,776.28	27,386.50
10	24	32	16	26,420.44	8815.35	18,471.25	20,035.25
11	26	30	14	25,712.33	8815.35	20,347.14	16,359.62
12	28	28	12	24,983.94	8815.35	23,654.18	12,684.00
13	30	26	10	24,564.34	8815.35	24,987.86	9008.37
14	32	24	8	23,571.95	8815.35	26,841.33	5332.74
15	34	22	5	25,610.65	8815.35	27,441.10	1657.12

The value of $\min _{i \in S^{*}} MID_{i}$ varies in the range of 23,342.55 to 29,365.05, which indicates a dispersion level of 19.73% of Pareto-front solutions in the optimal space based on different values of robustness parameters. Different combinations of robustness parameters for each objective function results in solutions which have significant differences (about 20%) in terms of MID. As it is complicated for ISIPO managers to agree on a final cluster for implementation in the province, it is necessary to investigate the sensitivity of objective function values based on different combinations of robustness parameters.

**Fig. 8:**
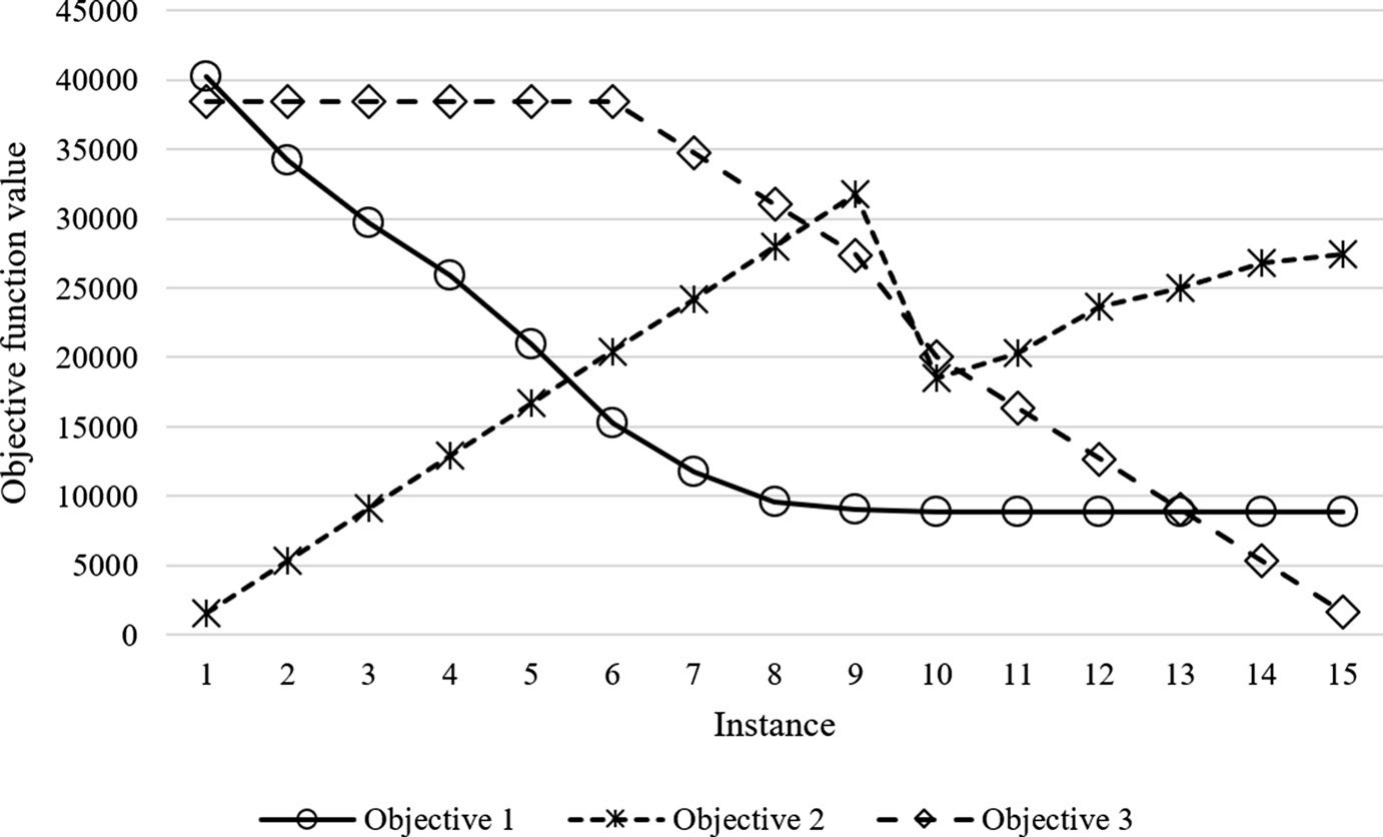
Sensitivity of objective functions to robustness parameters

In general, when increasing $\Gamma$, the value of the corresponding objective function will decrease, as one must hedge against a larger portion of the total uncertainty. However, when $\Gamma$ has reached a certain point, any further increase leads to unchanged solutions, as all relevant uncertainty has already been taken into account. Figure 8 graphically illustrates the sensitivity of different objective functions to the robustness parameters.

For $\Gamma _{1}>22$, the value of the first objective function remains unchanged, while for $\Gamma _{3}>24$, the third objective function is stable. For the second objective function, a similar stable level is never reached. Table 9 shows that combination of $\Gamma _{1}=12$, $\Gamma _{2}=14$ and $\Gamma _{3} = 28$ (instance 4) provides the best MID value, with objective function values $Z_{1}^{MID}=25,910.92$, $Z_{2}^{MID}=12,883.16$ and $Z_{3}^{MID}=38,413.38$. Table 10 shows the optimal assignment of government budget related to this Pareto-optimal solution. The largest shares of the total budget are assigned to the action plans P2 and P4 with 14%, P3 with 13%, P8 and P9 with 12%. Moreover, 27% of government incentives is in the form of providing cash payments (M4), and 38% of the incentives is about reducing or eliminating asset tax of enterprises (M2). These figures are similar to those in Table 8. The proposed framework not only effectively evaluates potential industrial clusters but also determine the optimal assignment of government budget to the selected cluster development plans.

**Table 10:** Optimal assignment of government budget to each action plan for the smallest value of MID

USD	P1	P2	P3	P4	P5	P6	P7	P8	P9	Sum
M1							6013			6013
M2	2547				517	8261			8500	26,825
M3		9996						8319		18,315
M4			9157	9982						19,139
**Sum**	3547	9996	9157	9982	6517	8261	6013	8319	8500	70,292

## Managerial insights

According to the results obtained in Sect. 6, the barberry and jujube industrial cluster is selected as one of the most important factors of economic development in the South Khorasan province. This selection is exactly in line with priorities of managers and decision makers in the province because helping businesses that are active in the field of strategic agricultural products, including barberry and jujube, has always been one of the most important operational plans in the province. Developing export, agro-food industries, planting and harvesting conditions, as well as improving processing infrastructure and creating added value are among the plans. Currently, about 97% of the world’s seedless barberry is planted by approximately 60% of the farmers in the province. The cities of Ghaen, Zirkuh, Birjand, and Darmian are known as the most important centers of barberry production in South Khorasan. From a perspective of export development, on average, only about 2% of the total barberry produced in the province enters foreign markets, which is also without processing and has the least added value. Therefore, planning to develop barberry export infrastructure can improve economic conditions in this area. This infrastructure involves various sectors of advertising, participating in international exhibitions, forming export consortia, formulating and editing export-related laws, producing organic products, and conducting studies to understand international markets.

Agro-food industries generally have not developed in spite of their great potential in the province; there is only one active concentrate barberry juice plant and its capacity is less than 5% of the barberry production. Moreover, barberry can be used in pharmaceutical products, but the necessary infrastructure for this requires joint ventures between the government and the private sector. Based on the results of the previous section, the action plans for marketing and trade development and for investment and financial planning receive the largest share of the total budget assigned to the barberry and jujube cluster. These two action plans can directly affect the improvement of export conditions and the development of agro-food industries. Therefore, the proposed model can be used as a suitable tool to assign financial resources.

From a technological point of view, all the steps of planting and harvesting are currently conducted using large amounts of pesticides, due to farmers’ lacking knowledge in how to produce without synthetic chemicals. Therefore, the development of an educational infrastructure, including cooperation agreements with knowledge-based centers to produce modern equipment for harvesting and storing barberries, as well as cooperation with educational and scientific institutions to inform farmers of new practical methods, can be important solutions.

From the perspective of developing group cooperation and creating production and sales networks, there is no systematic structure and all market participants operate individually and on a small scale. Thus, there can be some people who act as brokers, buying barberry from farmers at a low price and bringing the same product to the market at a higher price without any added value. Therefore, the development of cooperation networks between farmers, exporters and processing companies can increase the share of producers in the final price of the product in the market.

Finally, a weakness of management in various sectors of production, pricing, and sales in internal and external markets can be identified. Although the Agriculture Jihad Organization and the Chamber of Commerce, Industries, Mines, and Agriculture are responsible for managing the trends in barberry and jujube businesses, they perform poorly. For instance, the outbreak of the Corona virus in 2020 prevented many producers from selling their products, and barberry prices fell by 50%, severely affecting the economic situation of farmers who did not receive any government support. Therefore, creating flexible sales channels and resilient production and sales chains can play an important role in controlling critical situations.

Based on the results of the proposed mathematical model, implementation of action plans including cluster management system development (P3) with 13%, information technology development (P6), infrastructure development (P8) and raw materials planning (P9), each with 12% of the total cluster development budgets, can be used as appropriate solutions. The fact that cash payments receive a 24% share of the total budget shows that cash loans cannot be the main solution to the problems; however, a large part of government support programs are currently in the form of cash loans. The high share of financial resources to reduce asset tax and sales tax of enterprises confirms the existence of strict laws in the tax institutions as a major obstacle to the development of barberry and jujube cluster. If some laws are amended by the government, it can be a great help to solve the existing problems. In fact, in many sectors there is no need to use financial resources directly, and the government can help SMEs development simply by amending the laws.

## Conclusion

In this paper, the prioritization of industrial clusters and the assignment of governmental funding are investigated as a decision-making problem in two phases. In the first phase, a set of criteria and sub-criteria related to development of SMEs and industrial clusters are studied to prioritize different industrial clusters in the South Khorasan province of Iran. For this purpose, BWM is applied to determine weights of the criteria. Based on the obtained results, international market share attraction is the most important criterion, while the age of the cluster is the least important. Then, industrial clusters are prioritized by the VIKOR method. After performing numerical calculations, the barberry and jujube cluster is selected as the best option. In the second phase, a multi-objective mathematical model is designed to assign financial resources to various action plans for the development of industrial clusters. After solving the proposed model using CPLEX, 71 Pareto-optimal solutions are obtained, each of which can be used as the final solution. To select the top Pareto-front solution based on decision makers’ views, an innovative approach based on the mean ideal distance is presented to select one of the solutions for the final implementation.

## Supplementary Information

Below is the link to the electronic supplementary material.

Supplementary file 1 (docx 93 KB)

Supplementary file 2 (rar 67 KB)
