# Supplementary material for: Multiple criteria decision making and robust optimization to design a development plan for small and medium-sized enterprises in the east of Iran
Source: Oper Res Int J. 2023 Feb 25;23(1):13. doi: 10.1007/s12351-023-00761-1 (PMC9959954; doi:10.1007/s12351-023-00761-1)
Supplement: Supplementary file 1 — (docx 93 KB) [file 12351_2023_761_MOESM1_ESM.docx]

**Best-worst method**

BWM is one of the most powerful techniques of solving MCDM problems that can be used to gain weights of criteria (Rezaei, 2015, 2016). This method compensates for the weaknesses of pairwise comparison-based methods (such as AHP and ANP) like incompatibility. Additionally, the number of paired comparisons can be significantly reduced only by performing reference comparisons. BWM has been recently employed by researchers in different applications. In general, the structure of BWM consists of the following steps:

**Step 1.** Determine a set of decision criteria.

The criteria $\{c_{1}, c_{2}, \ldots,c_{n}\}$ have been obtained through literature research and experts’ opinions. These criteria can reflect performance of the alternatives.

**Step 2.** Determine the best and the worst criteria and sub criteria.

The best criterion $c_{B}$ and the worst one $w_{B}$ should be determined by the experts.

**Step 3.** Determine the reference comparison of the best criterion

The preference of the best criterion over the other criteria is determined by a number between 1 and 9 based on linguistic scales. The resulting vector is:

| (1) | $A_{B}=(a_{B1},a_{B2}, \ldots, a_{Bn})$ |
| --- | --- |

where $a_{Bj}$ is the preference of the best criterion B over criterion j. It is obvious that $a_{BB}=1$.

**Step 4.** Determine the preference comparison of the worst criterion.

The preference of the other criteria over the worst criterion is determined by a number between 1 and 9. The resulting vector is:

| (2) | $A_{w}=\left( a_{1W},a_{2W}, \ldots, a_{nW} \right)^{T}$ |
| --- | --- |

where $a_{jW}$ is the preference of the other criteria over the worst criterion. It is obvious that $a_{WW}=1$.

**Step 5.** Determine the optimal weights of criteria.

The maximum absolute differences $\left\{ \left| w_{B}-a_{Bj}w_{j} \right|,\left| w_{j}-a_{jW}w_{W} \right| \right\}$ for all j should be minimized to determine the optimal weights $(W_{1}^{*},W_{2}^{*},\ldots,W_{n}^{*})$. It can be formulated as the following optimization problem.

| (3) | $\min\max_{j} \left\{ \left\vert w_{B}-a_{Bj}w_{j} \right\vert,\left\vert w_{j}-a_{jW}w_{W} \right\vert\right\}$ |
| --- | --- |
|  | $S.t.$ |
|  | $\sum_{j} w_{j}=1$ |
|  | $w_{j}\geq0, for all j$ |

Problem 3 can be transferred to the following linear formulation.

| (4) | $\min\xi^{L}$ |
| --- | --- |
|  | $S.t.$ |
|  | $\left\vert w_{B}-a_{Bj}w_{j} \right\vert\leq\xi^{L}, for all j$ |
|  | $\left\vert w_{j}-a_{jW}w_{W} \right\vert\leq\xi^{L}, for all j$ |
|  | $\sum_{j} w_{j}=1$ |
|  | $w_{j}\geq0, for all j$ |

Problem 4 is linear with a unique solution. The optimal weights $(w_{1}^{*},w_{2}^{*},\ldots,w_{n}^{*})$ and the optimal value $\xi^{L*}$ will be obtained by solving the problem. Closer values of $\xi^{L*}$ to zero represent a high level of consistency (Rezaei, 2015).

**VIKOR technique**

VIKOR is a compromise ranking technique and is often applied in problems with conflicting criteria (Opricovic, 1998). This method produces a compromise solution based on closeness to the ideal solution and mutual agreement through concessions. This method has been used by many researchers to rank alternatives (Gupta, 2018). The steps of VIKOR are as follows.

**Step 1.** Determine a pairwise matrix for each alternative, and each criterion is evaluated using the linguistic scales.

**Step 2.** Calculate the average decision matrix using equation 5.

| (5) | $f_{ij}=\frac{1}{k}\sum_{t=1}^{k} x_{ij}^{t} i=1,2,\ldots,m;j=1,2,\ldots,n$ |
| --- | --- |

where $x_{ij}^{t}$ is the value of alternative i with respect to criterion j by expert t.

**Step 3.** Calculate the best $f_{j}^{*}$ and the worst $f_{j}^{-}$ values of all criteria using equations 6 and 7.

| (6) | for benefit criteria | $f_{j}^{*}=maxf_{ij}, i=1,2,\ldots,m;j=1,2,\ldots,n$  $f_{j}^{-}=minf_{ij}, i=1,2,\ldots,m;j=1,2,\ldots,n$ |
| --- | --- | --- |
| (7) | for cost criteria | $f_{j}^{*}=minf_{ij}, i=1,2,\ldots,m;j=1,2,\ldots,n$  $f_{j}^{-}=maxf_{ij}, i=1,2,\ldots,m;j=1,2,\ldots,n$ |

where $f_{j}^{*}$ and $f_{j}^{-}$represent positive ideal solution and negative ideal solution for criterion j respectively.

**Step 4.** Calculate values of $S_{i}$ and $R_{i}$ for $i=1,2,\ldots,m$ using equations 8 and 9.

| (8) | $S_{i}=\sum_{j=1}^{n} w_{j}\frac{(f_{j}^{*}-f_{ij})}{(f_{j}^{*}-f_{j}^{-})}$ |
| --- | --- |
| (9) | $R_{i}=\max\left[ w_{j}\frac{\left( f_{j}^{*}-f_{ij} \right)}{\left( f_{j}^{*}-f_{j}^{-} \right)} \right]$ |

where $S_{i}$ represents the distance of alternative i from the positive ideal solution, $R_{i}$ represents the distance of alternative i from the negative ideal solution, and $w_{j}$ indicates the criteria weights determined through BWM.

**Step 5.** Calculate $Q_{i}$ using equation 10.

| (10) | $Q_{i}=v\left[ \frac{S_{i}-S^{*}}{S^{-}-S^{*}} \right]+\left( 1-v \right)\left[ \frac{R_{i}-R^{*}}{R^{-}-R^{*}} \right]$ |
| --- | --- |

where $S^{-}=\max_{i} S_{i}, S^{*}=\min_{i} S_{i}$, $R^{-}=\max_{i} R_{i}, R^{*}=\min_{i} R_{i}$. v indicates the weight of maximum set utility and it is taken as 0.5 in this study.

**Step 6.** Rank the alternatives using $Q_{i}$ values.

**Step 7.** Alternatives should be ranked based on minimum values of $Q_{i}$ subject to satisfying the following two conditions simultaneously.

Condition 1 (acceptance attribute): Alternative $A^{1}$ is selected if $Q\left( A^{2} \right)-Q(A^{1})\geq1/m-1$ where $A^{2}$ is the alternative with the second rank and $\left( m \right)$ is the total number of alternatives.

Condition 2 (acceptance stability in decision making): $A^{1}$ should also have the first rank based on $S_{i}$ or/and $R_{i}$ values.

**Step 8.** The alternative obtaining the minimum $Q_{i}$ will be ranked first.

**References**

Deng, J.-L. (1982). Control problems of grey systems. *Sys. & Contr. Lett., 1*(5), 288-294.

Gupta, H. (2018). Evaluating service quality of airline industry using hybrid best worst method and VIKOR. *Journal of Air Transport Management, 68*, 35-47.

Opricovic, S. (1998). Multicriteria optimization of civil engineering systems. *Faculty of Civil Engineering, Belgrade, 2*(1), 5-21.

Rezaei, J. (2015). Best-worst multi-criteria decision-making method. *Omega, 53*, 49-57.

Rezaei, J. (2016). Best-worst multi-criteria decision-making method: Some properties and a linear model. *Omega, 64*, 126-130.
